# Application of a risk score model based on glycosylation-related genes in the prognosis and treatment of patients with low-grade glioma

**DOI:** 10.3389/fimmu.2024.1467858

**Published:** 2024-10-09

**Authors:** Binbin Zou, Mingtai Li, Jiachen Zhang, Yingzhen Gao, Xiaoya Huo, Jinhu Li, Yimin Fan, Yanlin Guo, Xiaodong Liu

**Affiliations:** ^1^ School of Basic Medical Sciences, Shanxi Medical University, Taiyuan, Shanxi, China; ^2^ Department of Neurosurgery, The First Hospital of Shanxi Medical University, Taiyuan, Shanxi, China

**Keywords:** low grade glioma, prognostic characteristics, glycosylation, immunotherapy, tumor immune microenvironment

## Abstract

**Introduction:**

Low-grade gliomas (LGG) represent a heterogeneous and complex group of brain tumors. Despite significant progress in understanding and managing these tumors, there are still many challenges that need to be addressed. Glycosylation, a common post-translational modification of proteins, plays a significant role in tumor transformation. Numerous studies have demonstrated a close relationship between glycosylation modifications and tumor progression. However, the biological function of glycosylation-related genes in LGG remains largely unexplored. Their potential roles within the LGG microenvironment are also not well understood.

**Methods:**

We collected RNA-seq data and scRNA-seq data from patients with LGG from TCGA and GEO databases. The glycosylation pathway activity scores of each cluster and each patient were calculated by irGSEA and GSVA algorithms, and the differential genes between the high and low glycosylation pathway activity score groups were identified. Prognostic risk profiles of glycosylation-related genes were constructed using univariate Cox and LASSO regression analyses and validated in the CGGA database.

**Results:**

An 8 genes risk score signature including ASPM, CHI3L1, LILRA4, MSN, OCIAD2, PTGER4, SERPING1 and TNFRSF12A was constructed based on the analysis of glycosylation-related genes. Patients with LGG were divided into high risk and low risk groups according to the median risk score. Significant differences in immunological characteristics, TIDE scores, drug sensitivity, and immunotherapy response were observed between these groups. Additionally, survival analysis of clinical medication information in the TCGA cohort indicated that high risk and low risk groups have different sensitivities to drug therapy. The risk score characteristics can thus guide clinical medication decisions for LGG patients.

**Conclusion:**

Our study established glycosylation-related gene risk score signatures, providing new perspectives and approaches for prognostic prediction and treatment of LGG.

## 1 Introduction

Glioma is one of the most common tumors in the human central nervous system (CNS), characterized by the origin in glial cells of CNS (1). It is known for its rapid growth and aggressive tendencies (2). WHO grade II gliomas, such as diffuse astrocytomas and oligodendrogliomas, are classified as low-grade glioma (LGG) (3) (4). These tumors usually have a long and slow course of disease. However, studies have shown that LGG may undergo malignant transformation, leading to severe disability and death (5), thus significantly reducing the quality of life and survival rates of patients (6). Although conventional surgical resection, radiotherapy combined with immunotherapy, and novel electric field therapy have improved the prognosis of glioma patients, the overall prognosis remains poor due to the heterogeneity of LGG (7). Therefore, there is an urgent need for a comprehensive understanding of the molecular mechanisms underlying glioma genesis and development, the discovery of new biomarkers, and the improvement and validation of innovative predictors to accurately assess the prognosis of patients with LGG.

Glycosylation is an enzymatic process that involves linking sugars to proteins, lipids, and other glycans. This major post-translational modification (PTM) occurs in the endoplasmic reticulum and Golgi apparatus of all cells and is mediated by the coordinated action of different glycosyltransferases and glycosidases (8). Glycosylation-mediated post-translational modifications play a crucial role in regulating fundamental processes such as cell division, differentiation, immune response, and cell-cell interactions. Altered N-linked or O-linked glycosylation patterns of regulatory proteins, such as transcription factors or cellular receptors, contribute to a variety of diseases, including cancer. These alterations contribute to microscopic and macroscopic heterogeneity of tumor cells (9). Studies have shown that glycosylation-related genes are strongly associated with the prognosis of patients with breast cancer (10), ovarian cancer (11), liver cancer (12), cervical cancer (13), and pancreatic cancer (14). Tumor cells generally exhibit abnormal glycosylation patterns compared to non-malignant cells. Abnormally expressed glycosylation-related genes have been shown to be potent biomarkers for a variety of tumors (15).

Therefore, it is essential to delve into the analyzing the expression and prognosis of glycosylation-related genes in LGG, and constructing the prognosis model by the glycosylation-related genes for optimizing the diagnosis, prevention, and management of LGG.

## 2 Materials and methods

### 2.1 Data collecting

We obtained transcriptome data and clinical information for low-grade glioma (LGG) from the Cancer Genome Atlas (TCGA) database (https://portal.gdc.cancer.gov/) using the “TCGAbiolinks^”^ (16) R package, including data from a total of 504 LGG patients with survival information. Additional LGG validation data were sourced from the CGGA-LGG693 and CGGA-LGG325 datasets in the China Glioma Genome Atlas (CGGA) database (http://www.cgga.org.cn), where we selected patients classified as WHO grade II, resulting in 172 patients in the CGGA-LGG693 cohort and 98 patients in the CGGA-LGG325 cohort. Furthermore, single-cell transcriptomic data (GSE117891) (17) were collected from the Gene Expression Omnibus (GEO, https://www.ncbi.nlm.nih.gov/geo/), encompassing samples from 73 regions in 13 patients with glioma and 1 patient with brain metastases. Gene expression data from 1,152 cases of normal brain tissue were obtained from the Genotype-Tissue Expression (GTEx) project (https://commonfund.nih.gov/GTEx). For the LGG pediatric peptide vaccine immunotherapy cohort (12 patients) versus the GBM (Glioblastoma) anti-PD-1 immunotherapy cohort (34 patients), data were sourced from the TIGER database (http://tiger.canceromics.org/).

### 2.2 Single-cell RNA sequencing analysis

We utilized the R package “Seurat” (18) for the analysis of single-cell RNA sequencing (scRNA-seq) data from Glioma patients. Initially, we conducted quality control measures, which included filtering out low quality genes detected in fewer than three cells, removing low-quality cells with fewer than 300 identified genes, excluding cells with mitochondrial gene content greater than 15%, ribosomal gene content less than 3%, and hemoglobin gene content greater than 0.1%. We also filtered out the MALAT1 housekeeping gene and mitochondrial genes, and removed doublets. For the retained cells, we normalized the gene expression matrix using the NormalizeData function in Seurat, and applied centering and scaling using the ScaleData function. We extracted the top 2000 highly variable genes and then performed principal component analysis (PCA), utilizing the top 20 principal components for clustering. The cell populations were visualized using Uniform Manifold Approximation and Projection (UMAP). Clustering analysis was performed using the FindClusters function with a resolution of 0.3 to identify distinct cell clusters.

To identify cell types, we employed specific cell markers sourced from the official CellMarker 2.0 website (http://117.50.127.228/CellMarker/). The FindAllMarkers function in Seurat was used to determine differentially expressed genes across various cell clusters.Using the default method of the FindAllMarkers function, the Wilcoxon rank-sum test, we screened for differentially expressed genes in the microglia population, selecting those with p.adjust < 0.05 and an absolute log2FC > 0.585.

### 2.3 Glycosylation gene sets enrichment score of cell clusters

To calculate the glycosylated gene sets enrichment score within each cell cluster, we employed the irGSEA.score function from the “irGSEA” (19) package. The KEGG glycosylated gene sets (C2 classification), including “KEGG_N_GLYCAN_BIOSYNTHESIS” and “KEGG_O_GLYCAN_ BIOSYNTHESIS”, was downloaded from the MSigDB database, and several enrichment analysis algorithms were applied to normalized RNA sequencing data, including AUCell, UCell, and ssGSEA.

### 2.4 Glycosylation pathway activity analysis

We computed the KEGG glycosylation pathway enrichment score for each patient in the TCGA-LGG cohort using GSVA (20). Patients were divided into high and low N-glycosylation groups based on the median N-glycosylation score, and similarly into high and low O-glycosylation groups based on the median O-glycosylation score. Differential expression analysis was performed using “Deseq2” (21), with thresholds of P.adjust < 0.05 and the absolute value of log2FC > 0.585 to identify differentially expressed genes. These genes were used to investigate the molecular characteristics associated with high and low activity in the N-glycosylation and O-glycosylation pathways.

### 2.5 Functional enrichment analysis

Gene Ontology (GO) and Kyoto Encyclopedia of Genes and Genomes (KEGG) functional enrichment analyses were conducted on the differentially expressed genes using the R package “clusterProfiler” (22). Only results with p.adjust < 0.05 were considered significantly enriched.

### 2.6 Immune infiltration analysis

The ESTIMATE (23) (Estimation of Stromal and Immune Cells in Malignant Tumors Using Expression Data) algorithm was utilized to assess changes in immune scores, stromal scores, ESTIMATE scores, and tumor purity in the samples. Additionally, the relative abundance of various cell types was determined using the “CIBERSORT” (24) and “GSVA” (20) R packages, employing the CIBERSORT and ssGSEA (Single Sample Gene Set Enrichment Analysis) methods, respectively.

### 2.7 Prognostic glycosylation based signature construction

To establish a glycosylation-based prognostic signature, we performed univariate Cox regression and least absolute shrinkage and selection operator (LASSO) regression with 10-fold cross-validation using the R package “glmnet” (25). In the LASSO regression, we selected “lambda.min” to prevent overfitting. The risk score for each LGG patient was calculated using the following formula:

Risk score = (0.382 × ASPM expression) + (0.017 × CHI3L1 expression) + (0.068 × LILRA4 expression) + (0.258 × MSN expression) + (0.116 × OCIAD2 expression) + (-0.137 × PTGER4 expression) + (0.109 × SERPING1 expression) + (0.073 × TNFRSF12A expression)

### 2.8 Validation and performance evaluation

Kaplan-Meier analysis compared overall survival between high and low risk score groups, stratified by the median risk score. Receiver Operating Characteristic (ROC) curves generated with the “timeROC” (26) R package assessed the predictive accuracy of risk score for 1-, 3-, and 5-year survival.

### 2.9 Prognostic nomogram construction

Prognostic nomograms incorporating risk score and other clinical features were constructed using the R package “rms”. The performance of these nomograms was evaluated through calibration curves and ROC curves to assess their predictive accuracy and reliability.

### 2.10 Prognostic feature gene analysis

We combined the expression data from the TCGA-LGG cohort with expression data from normal brain tissues in the GTEx database and performed a Wilcoxon test using the R package “rstatix”. Boxplots were then generated to visualize gene expression levels. Additionally, we calculated the proportion of each cell type in each sample from the single-cell data and determined the average gene expression within each cell type group. The gene with the highest average expression was used to compute the correlation between average gene expression and cell content in each sample, using the cor.test function from base R. The cor.test function employed Pearson’s correlation coefficient to assess the linear relationship between gene expression and cell content.

### 2.11 Prediction of immunotherapy responsiveness

The Tumor Immune Dysfunction and Exclusion (TIDE) algorithm was utilized to predict the potential response of LGG patients to immune checkpoint inhibitors (ICIs) treatment (27). A higher TIDE score suggests a decreased likelihood of benefiting from immunotherapy and an increased risk of immune escape. The Dysfunction score indicates the level of immune cell dysfunction infiltrating the tumor, while the Exclusion score reflects the degree of immune cell exclusion within the tumor microenvironment.

### 2.12 Drug sensitivity analysis

To predict susceptibility to different drugs in LGG patients within the high and low risk score groups, drug treatment sensitivity was assessed using the R package “oncoPredict” (28). This analysis used expression matrices from the Cancer Drug Sensitivity Genomics (GDSC) database and drug treatment information as a training set. The half-maximal inhibitory concentration (IC50) served as the primary indicator for evaluating the sensitivity of LGG cancer cells drugs. Differences in IC50 between the high and low risk score groups were compared using the Wilcoxon test, with p < 0.05 considered statistically significant.

### 2.13 Statistical analysis

All statistical analyses were conducted using R software (version 4.3.0). The R packages employed in this study are open access and freely available. Statistical significance was determined at a threshold of p < 0.05, with levels indicated as follows: * p < 0.05; ** p < 0.01; *** p < 0.001.

## 3 Results

### 3.1 Annotation of cell types calculation the glycosylation scores and marker gene of the cell type

**Figure 1:**
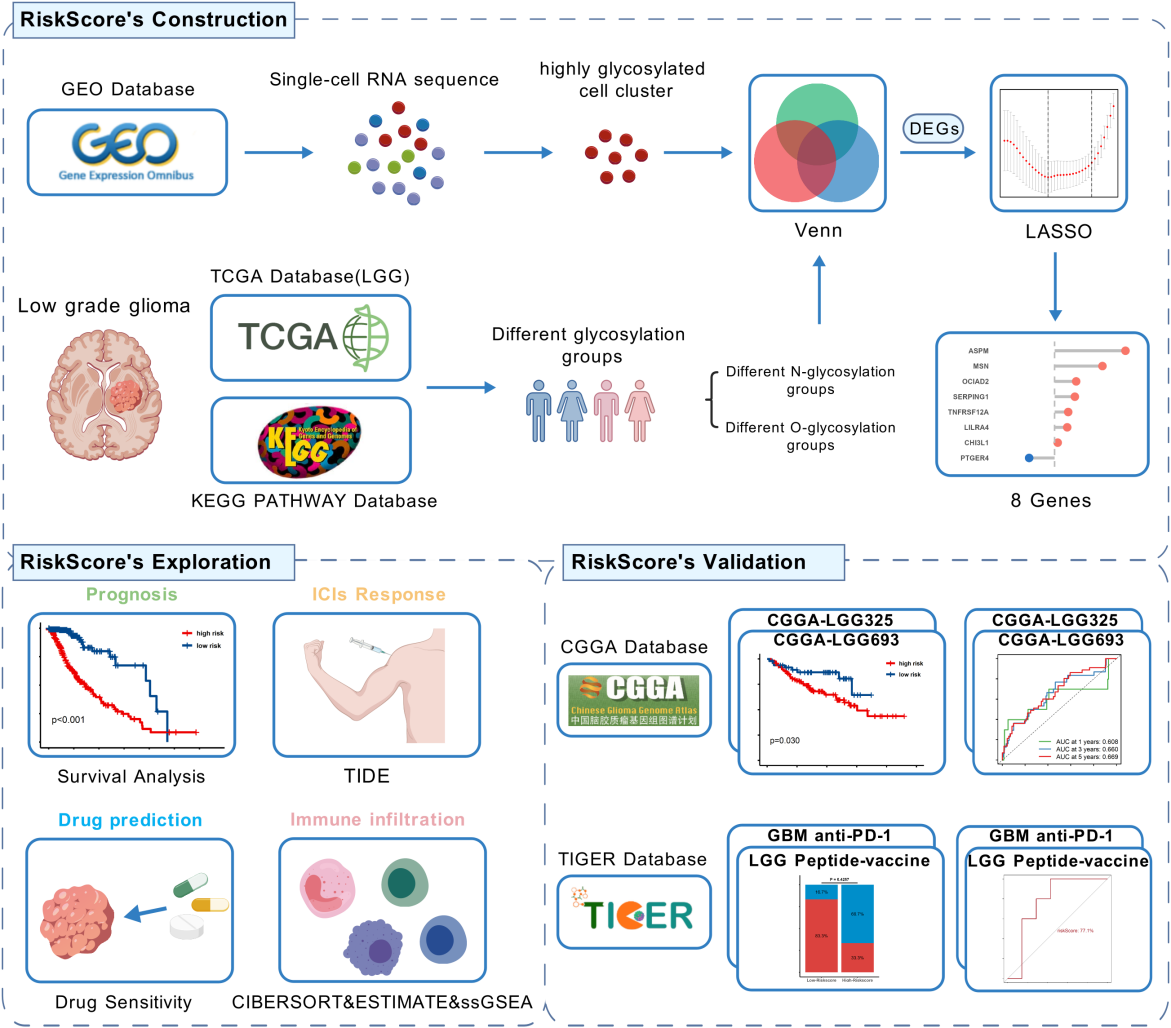
Flow chart of this study,created using BioGDP.com. (GEO, Gene Expression Omnibus; TCGA, The Cancer Genome Atlas; CGGA, The China Glioma Genome Atlas; LGG, low-grade glioma; GBM, Glioblastoma; LASSO, Least Absolute Shrinkage and Selection Operator; DEGs, Differentially Expressed Genes; TIDE, Tumor Immune Dysfunction and Exclusion).

**Figure 2:**
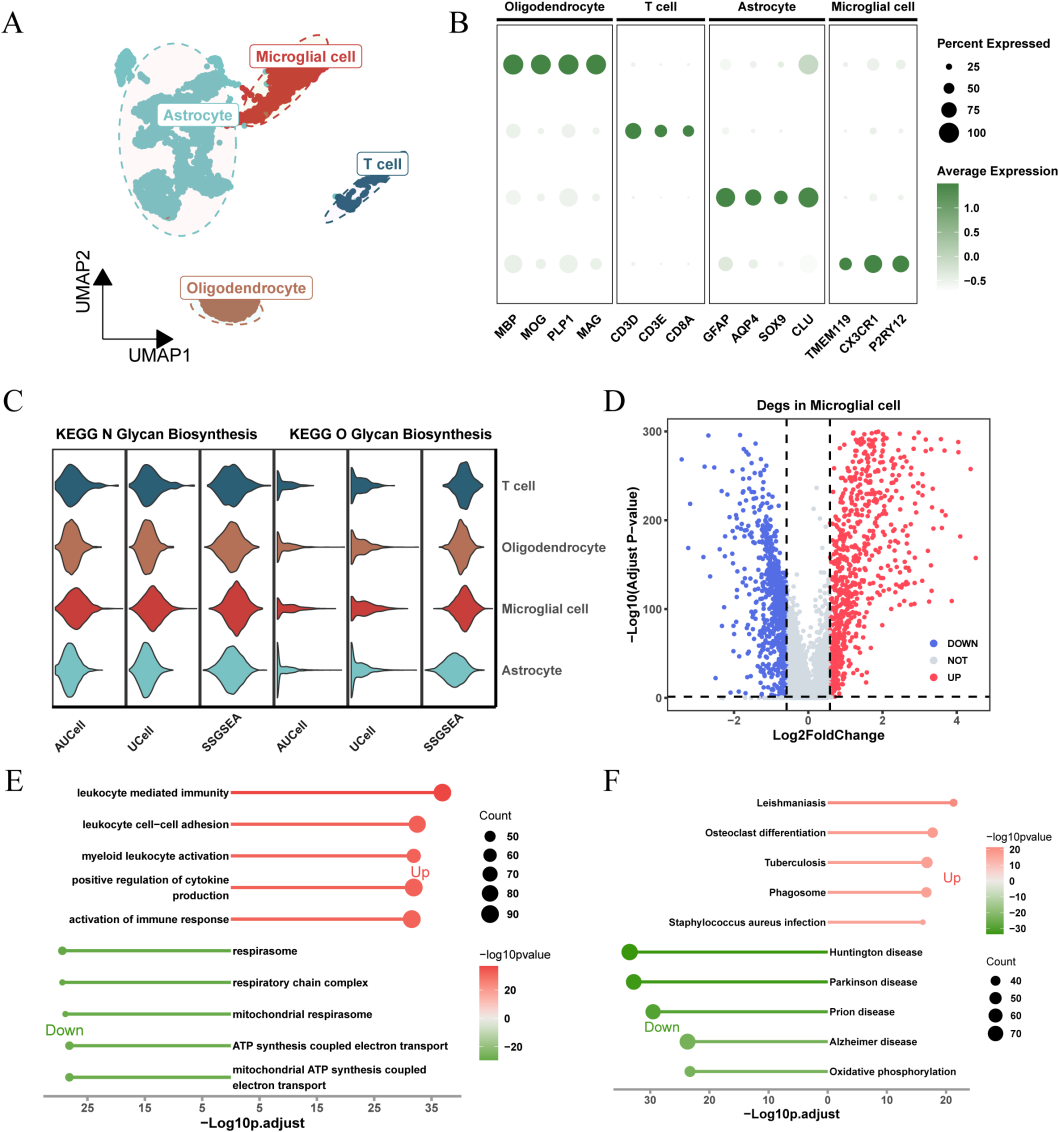
Analysis of glycosylation pathway in single-cell RNA sequencing of glioma and its biological significance. **(A)** UMAP plots illustrating the distribution of four major cell types within the comprehensive dataset. **(B)** Dot plot depicting marker gene expression levels across each identified cell type. **(C)** Expression and distribution of N-glycosylation and O-glycosylation pathway activity scores across different cell types. **(D)** Volcano plot displaying differentially expressed genes (DEGs) comparing the microglial population versus other subpopulations (logFC > 0.585, P.adj < 0.05). **(E)** Gene Ontology (GO) analysis results, with -Log10 (adjusted p-value) plotted on the horizontal axis. **(F)** KEGG pathway analysis results, with -Log10 (adjusted p-value) plotted on the horizontal axis.

**Figure 1** shows the flow chart of this study. In the GSE117891 dataset, we analyzed a total of 5,368 cells and 22,559 genes. By collecting common cell marker genes for brain tissue from the CellMarker 2.0 website and combining them with classical cell markers, we ultimately annotated four cell types: oligodendrocyte (MBP, MOP, PLP1, MAG), T cell (CD3D, CD3E, CD8A), astrocyte (GFAP, AQP4, SOX9, CLU), and microglial cell (TMEM119, CX3CR1, P2RY12). Oligodendrocyte accounted for 744 cells, approximately 13.7%, T cell accounted for 379 cells, approximately 7.1%, astrocyte accounted for 2,993 cells, approximately 55.8%, and microglial cell accounted for 1,252 cells, approximately 23.4%. These cell types were visualized using UMAP and DotPlot (**Figure 2A** detailed in **Figure 2B** and **Supplementary Figure 1A**).

Based on the expression levels of genes from the “KEGG_N_GLYCAN_BIOSYNTHESIS” and “KEGG_O_GLYCAN_BIOSYNTHESIS” pathways in the KEGG pathway database, we calculated glycosylation scores for each cell type using three algorithms: AUCell, UCell, and ssGSEA. The results showed that, for both N-glycosylation and O-glycosylation, microglia exhibited the highest glycosylation scores across all three algorithms, significantly higher than other cell types (**Figure 2C**; **Supplementary Figure 1B**). This suggests that glycosylation may play an important role in the function of microglia.

To understand the unique molecular characteristics of microglia, we identified 1,684 differentially expressed genes (DEGs) compared to other cell types and visualized these genes using a volcano plot (**Figure 2D**). GO and KEGG enrichment results (**Figures 2E, F**) showed that these DEGs were enriched in pathways related to cytokine regulation, immune response activation, ATP synthesis coupled with electron transport, and other related signaling pathways.

### 3.2 TCGA-LGG cohort glycosylation analysis

**Figure 3:**
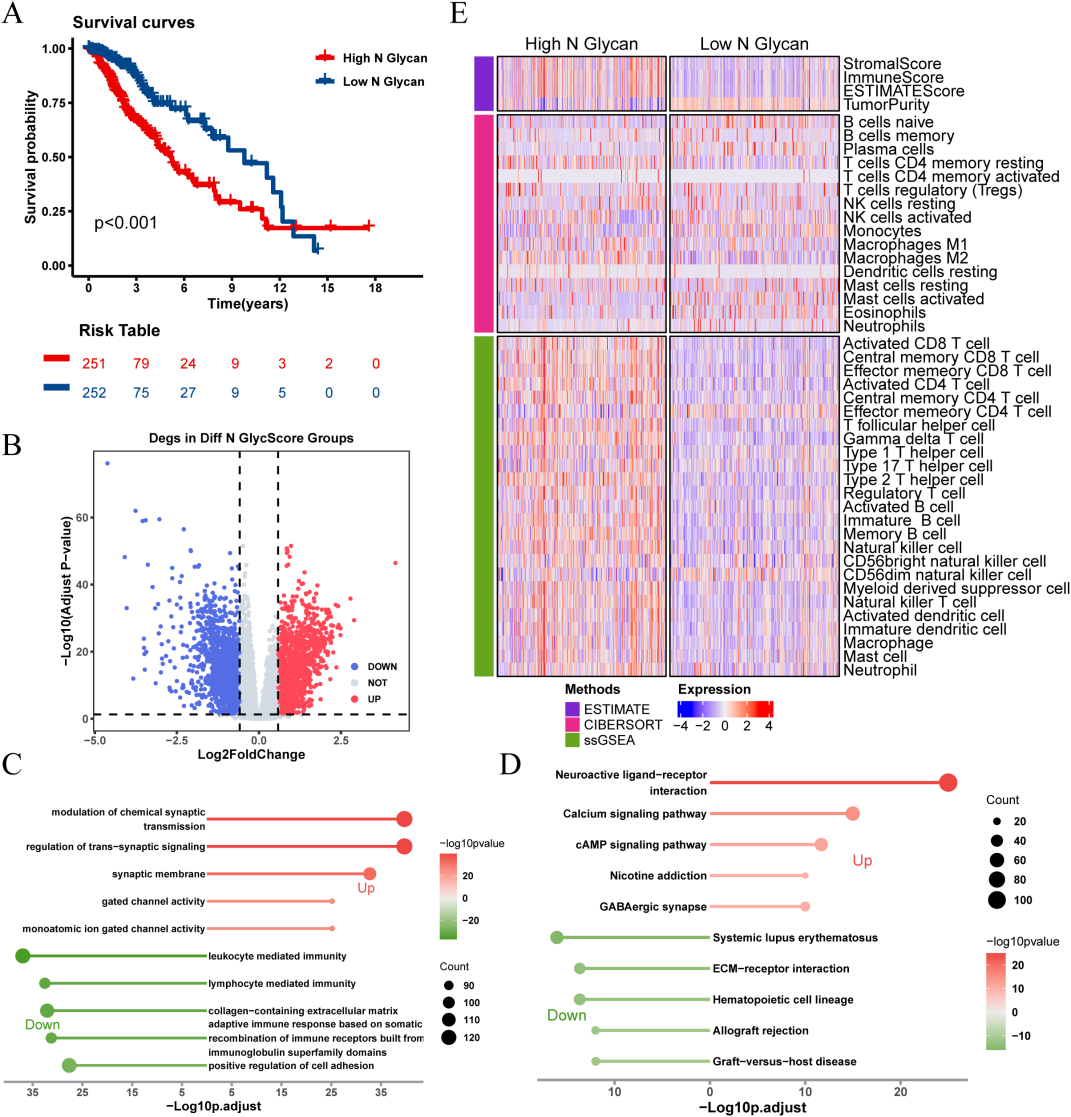
Analysis of N-glycosylation pathway in TCGA-LGG cohort and its biological significance. **(A)** Kaplan-Meier survival curves comparing overall survival between high and low N-glycosylation subgroups. **(B)** Volcano plot showing differentially expressed genes (DEGs) between high and low N-glycosylation subgroups (logFC > 0.585, P.adj < 0.05). **(C)** GO analysis results, plotted with -Log10 (adjusted p-value) on the horizontal axis. **(D)** KEGG pathway analysis results, plotted with -Log10 (adjusted p-value) on the horizontal axis. **(E)** Heatmap illustrating differences in immune scores between high and low N-glycosylation subgroups, highlighting variations in immune microenvironments.

Kaplan-Meier survival analysis revealed significant survival differences based on N-glycosylation pathway activities (P < 0.001, **Figure 3A**), indicating that patients with lower N-glycosylation pathway activity had better prognoses. We identified 3,019 DEGs between high and low N-glycosylation pathway activity subgroups (**Figure 3B**) and performed functional enrichment analyses using GO and KEGG, which highlighted potential alterations in immune signaling pathways (**Figures 3C, D**). Furthermore, we evaluated the immune microenvironment of each patient. Results indicated that patients with high N-glycosylation pathway activity exhibited elevated stromal scores, immune scores, and ESTIMATE scores, along with reduced tumor purity, indicative of increased tumor heterogeneity and a more complex immune microenvironment (**Figure 3E**).

**Figure 4:**
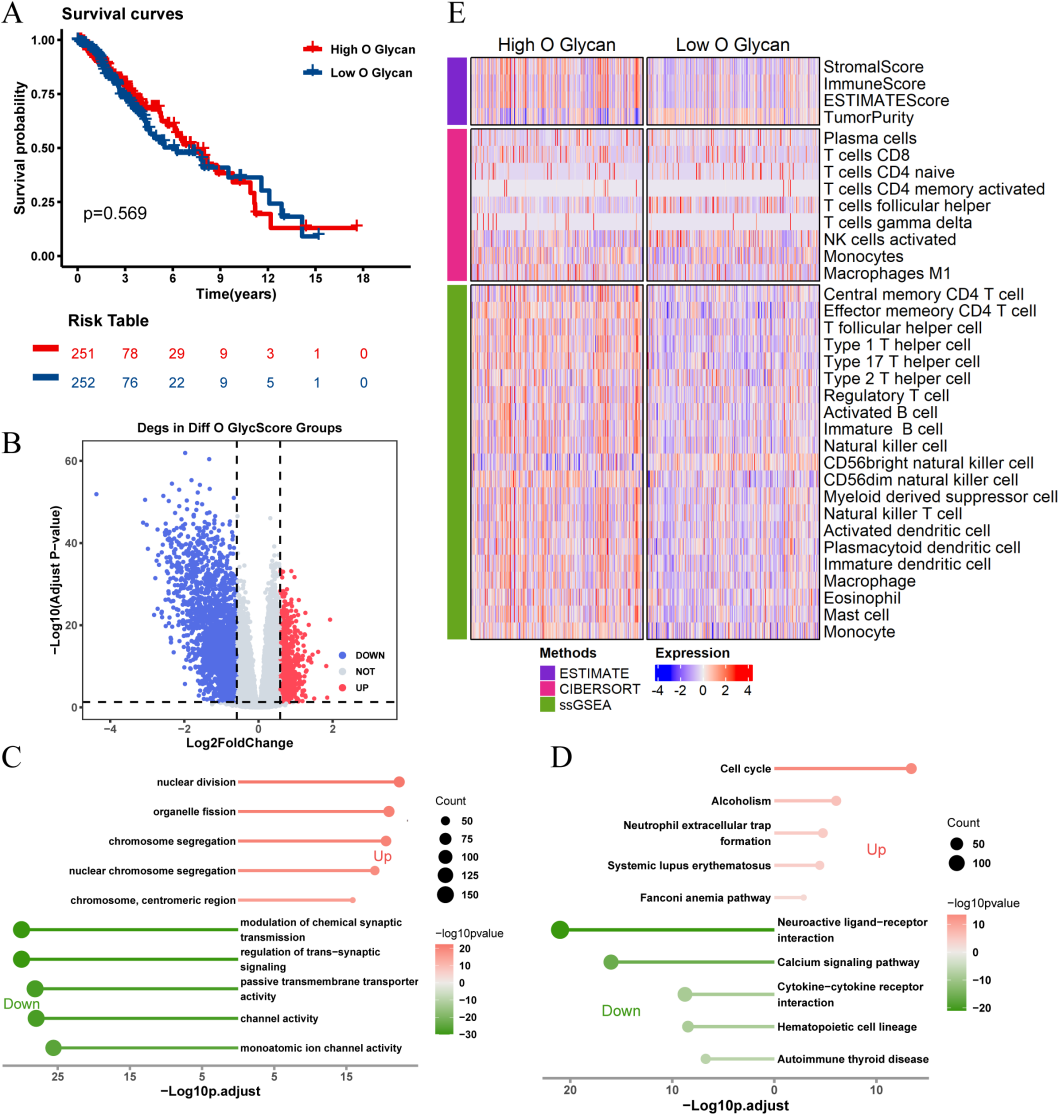
Analysis of O-glycosylation pathway in TCGA-LGG cohort and its biological significance. **(A)** Kaplan-Meier survival curves comparing overall survival between high and low O-glycosylation subgroups. **(B)** Volcano plot showing differentially expressed genes (DEGs) between high and low O-glycosylation subgroups (logFC > 0.585, P.adj < 0.05). **(C)** GO analysis results, plotted with -Log10 (adjusted p-value) on the horizontal axis. **(D)** KEGG pathway analysis results, plotted with -Log10 (adjusted p-value) on the horizontal axis. **(E)** Heatmap illustrating differences in immune scores between high and low O-glycosylation subgroups, highlighting variations in immune microenvironments.

There was no significant difference in prognosis between high and low O-glycosylation groups (**Figure 4A**). Similarly, enrichment analysis of 3,164 DEGs between high and low O-glycosylation pathway activity groups (**Figure 4B**) revealed that these genes were enriched in signaling pathways related to chromosome segregation, synaptic signaling regulation, and the cell cycle (**Figures 4C, D**). The high O-glycosylation pathway activity group showed higher matrix scores, immune scores, and ESTIMATE scores compared to the low-activity group, suggesting a similarly complex immune microenvironment (**Figure 4E**).These findings underscore the potential role of N-glycosylation and O-glycosylation levels in influencing immune cell types and responses within the LGG tumor microenvironment.

### 3.3 Establishing and validating glycosylation-based prognostic features

**Figure 5:**
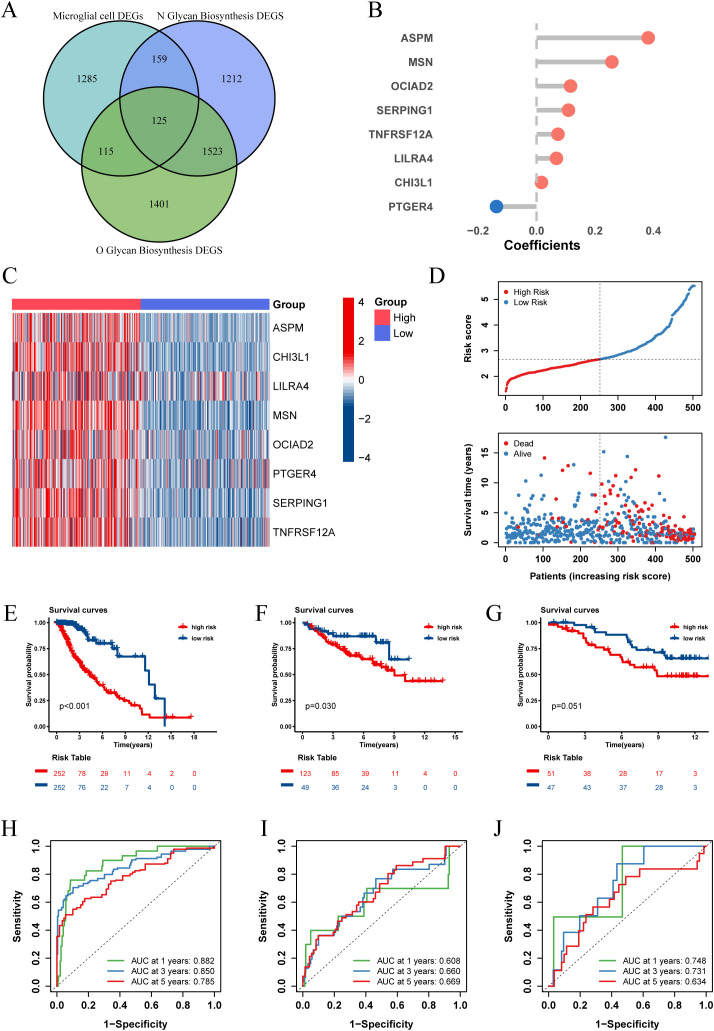
Establishment and validation of risk score. **(A)** The Venn diagram illustrates the intersection of differentially expressed genes (DEGs) between microglia and other cell types from the GSE117891 dataset, DEGs between high and low N-glycosylation groups in the TCGA-LGG cohort, and DEGs between high and low O-glycosylation groups in the same cohort. **(B)** Contribution coefficients of the individual constituent genes in the risk score model. **(C)** Heatmap showing the expression levels of the eight genes across different risk score subgroups in the TCGA-LGG cohort. **(D)** Distribution of risk score among patients in the TCGA-LGG cohort, ranked from lowest to highest. The survival status of each patient is classified according to their risk score. **(E-G)** Kaplan-Meier survival curves in three cohorts (TCGA-LGG, CGGA-LGG693, CGGA-LGG325), demonstrating the differences in overall survival between the high and low risk score subgroups. **(H-J)** ROC curves describing the predictive performance of risk score for 1-, 3-, and 5-year overall survival in patients with LGG in the three cohorts.

Recognizing the impact of glycosylation on the prognosis and immune microenvironment of patients with LGG, we further explored prognostic features to understand the potential complexity of LGG. We compared the DEGs of microglia in GSE117891 with other cell type, the DEGs of the high-low N-glycosylation subgroup in the TCGA-LGG cohort, and the DEGs of the high-low O-glycosylation subgroup, and identified 125 intersecting genes (**Figure 5A**). Through univariate Cox regression analysis, 105 of 125 genes were confirmed to be associated with prognosis. After LASSO regression narrowing, eight key genes were identified (**Supplementary Figures 2A, B**), constructing a prognostic feature model called risk score (**Figure 5B**), including ASPM, CHI3L1, LILRA4, MSN, OCIAD2, PTGER4, SERPING1, and TNFRSF12A.

We calculated the risk score for each patient in the TCGA-LGG cohort and divided the patients into high and low risk score groups based on the median risk score, showing the survival status plots of the patients. The results indicated that patients with higher risk score values exhibited increased expression levels of ASPM, CHI3L1, LILRA4, MSN, OCIAD2, PTGER4, SERPING1, and TNFRSF12A (**Figure 5C**), which were associated with poorer survival outcomes (**Figure 5D**). Kaplan-Meier analysis showed that overall survival (OS) was significantly better in the low risk score group (P < 0.001, **Figure 5E**).

To assess the predictive power of our prognostic features, we generated receiver operating characteristic (ROC) curves (**Figure 5H**) for 1-, 3-, and 5-year OS, with areas under the curve (AUC) of 0.882, 0.859, and 0.785, respectively, indicating good predictive performance of our model. Additionally, we validated our prognostic features in two independent external validation sets (CGGA-LGG693, CGGA-LGG325), yielding satisfactory results with 5-year AUCs of 0.669 and 0.634, respectively (**Figures 5F, G, I, J**).

**Figure 6:**
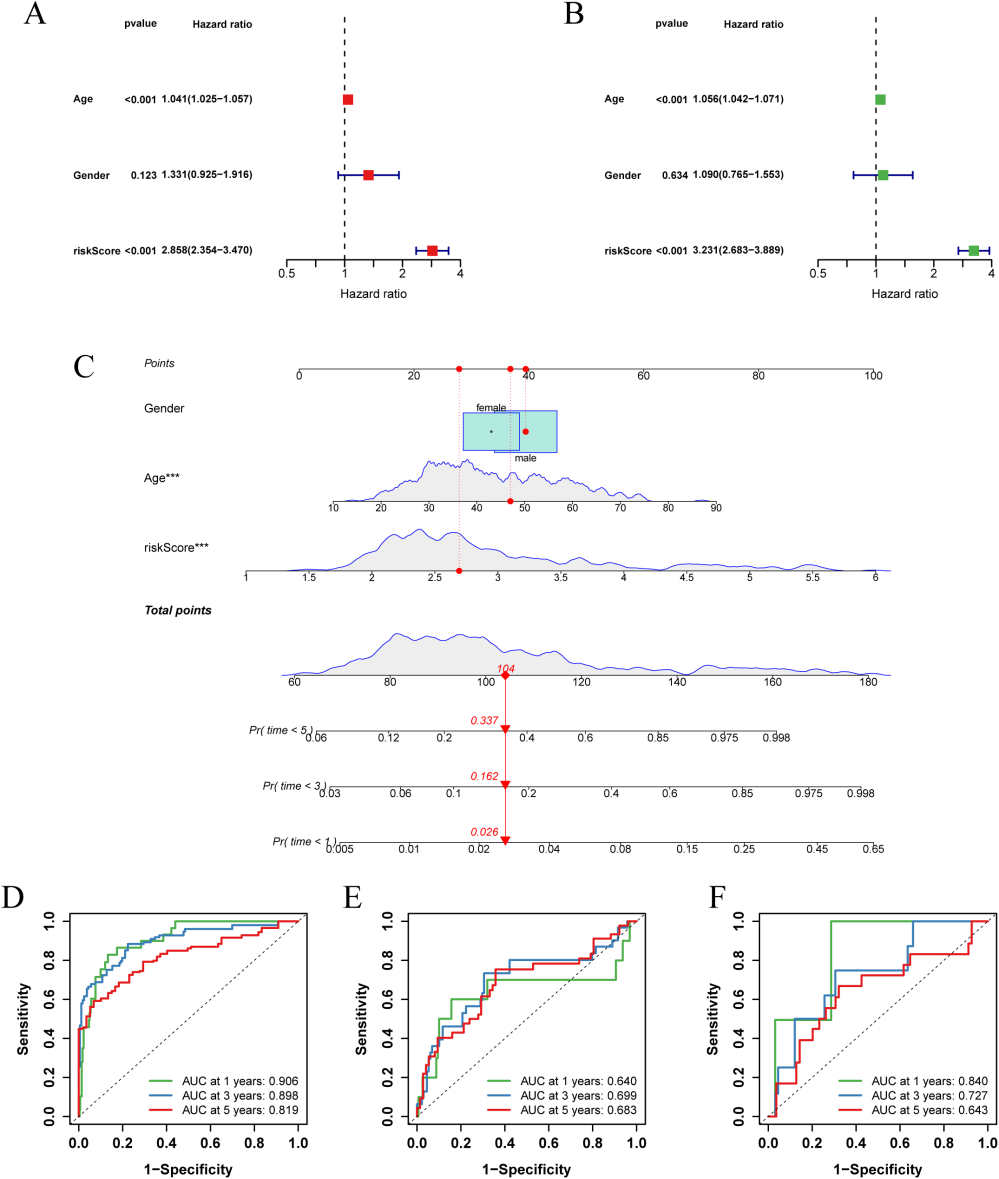
Establishment and validation of nomograms. **(A)** Univariate Cox regression analysis of clinical characteristics and risk score in the TCGA-LGG cohort. **(B)** Multivariate Cox regression analysis of clinical characteristics and risk score in the TCGA-LGG cohort. **(C)** Nomogram combining age, sex, and risk score for predicting 1-, 3-, and 5-year overall survival in LGG patients. **(D-F)** ROC curves describing the predictive performance of the nomograms for 1-, 3-, and 5-year overall survival in patients with LGG in three cohorts (TCGA-LGG, CGGA-LGG693, CGGA-LGG325).

To improve clinical utility, we created nomograms combining risk score and clinical characteristics. Univariate and multivariate Cox regression analyses, which assessed the effect of clinical characteristics and risk score on LGG survival, showed that age and risk score were important predictors of survival (**Figures 6A, B**). Based on these predictors, we designed a nomogram to estimate the 1-, 3-, and 5-year survival probabilities of patients with LGG (**Figure 6C**). The ROC curve showed that the nomogram had an AUC of 0.906 at 1 year, 0.898 at 2 years, and 0.819 at 3 years (**Figure 6D**), and the calibration curve demonstrated the predictive accuracy of the nomogram (**Supplementary Figure 2H**). In addition, we validated the predictive performance of nomograms in two independent external validation sets (**Figures 6E, F**; **Supplementary Figures 2I, J**).

Collectively, these results suggest that risk score-based nomograms, rooted in the concept of glycosylation, have substantial clinical predictive value for LGG.

### 3.4 Differences in the expression of prognostic feature genes and immune landscapes

**Figure 7:**
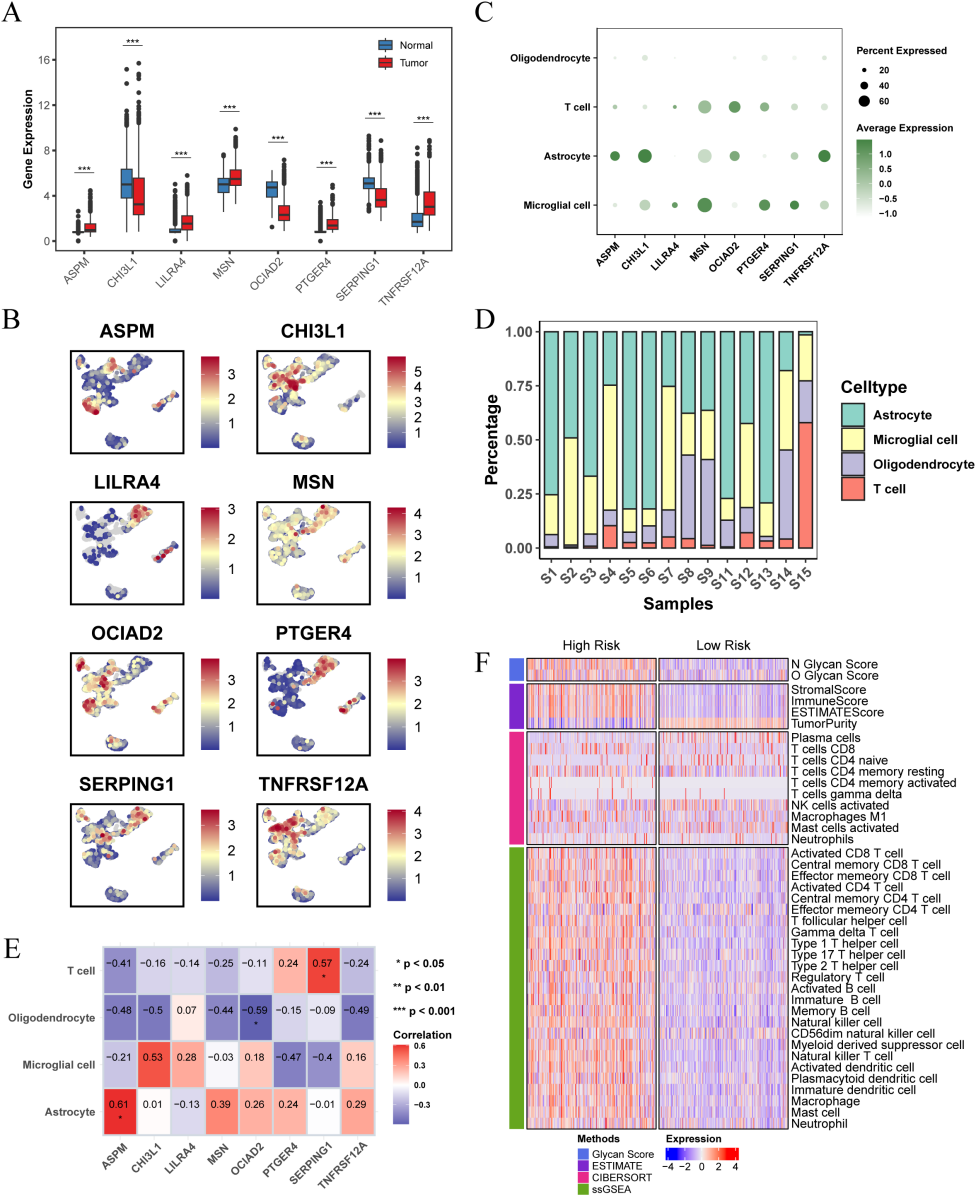
Differential expression of prognostic characteristic genes and immune microenvironment. **(A)** Differential expression of the 8 prognostic signature genes in normal brain tissue and LGG tissue, analyzed using data from the TCGA combined with the GTEx database. **(B)** Expression and distribution of the 8 prognostic signature genes across different cell types in the GSE117891 single-cell dataset. **(C)** Dot plot showing the expression levels of 8 prognostic signature genes across different cell populations. **(D)** Bar plot showing the percentage of each cell population within each sample in the GSE117891 single-cell dataset. **(E)** Heatmap showing the Pearson correlation between cell proportions and gene expression, with the numbers in the blocks representing correlation coefficients and asterisks indicating p-values. **(F)** Heatmap showing differences in immune scores and immune microenvironments between high and low risk score subgroups in the TCGA-LGG cohort. (* p < 0.05 ; ** p < 0.01; *** p < 0.001).

The analysis of the TCGA-LGG cohort combined with GTEx normal brain tissue data revealed notable expression differences of the prognostic feature genes. Specifically, ASPM, LILRA4, MSN, PTGER4, and TNFRSF12A were significantly upregulated in LGG patients compared to normal tissues, while CHI3L1, OCIAD2, and SERPING1 exhibited decreased expression (**Figure 7A**). We further examined the distribution of these eight genes in the GSE117891 dataset (**Figure 7B, C**). The results indicated that ASPM, CHI3L1, and TNFRSF12A were more highly expressed in astrocyte, while LILRA4, MSN, PTGER4, and SERPING1 were more highly expressed in microglial cell. OCIAD2 was found to be more highly expressed in T cell. We calculated the proportion of each cell type in each sample and determined the average expression of genes in the cell group with the highest expression. We then analyzed the correlation between gene expression and cell proportion (**Figure 7D, E**). The results indicated that ASPM expression was positively correlated with the proportion of astrocyte (cor = 0.61, p < 0.05), OCIAD2 expression was negatively correlated with the proportion of oligodendrocyte (cor = -0.59, p < 0.05), and SERPING1 expression was positively correlated with the proportion of T cell (cor = 0.57, p < 0.05).

We also investigated the differences in the immune microenvironment between patients in different risk score groups within the TCGA-LGG cohort. The findings showed a positive correlation between glycosylation score and risk score. Patients in the high risk score group had higher stromal, immune, and ESTIMATE scores compared to those in the low risk score group, but exhibited lower tumor purity (**Figure 7F**). This indicates a more complex immune microenvironment, characterized by increased tumor heterogeneity.

### 3.5 Response to immunotherapy in different risk score groups

**Figure 8:**
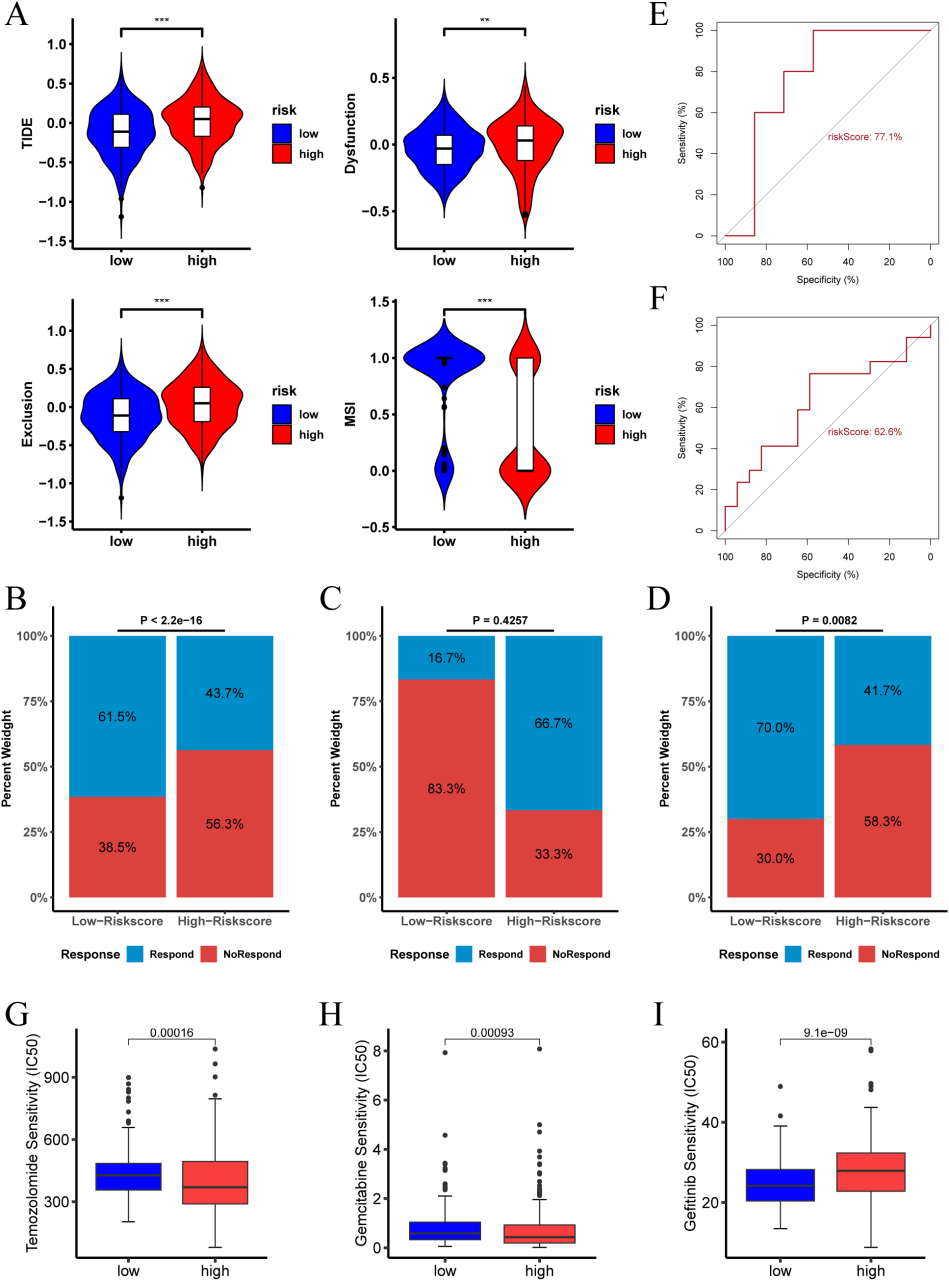
Immunotherapy response and drug sensitivity analysis of risk score. **(A)** TIDE score, Dysfunction score, Exclusion score, and MSI for different risk score groups in the TCGA-LGG cohort. **(B)** Proportion of immunotherapy responders in different risk score groups in the TCGA-LGG cohort. **(C)** Proportion of immunotherapy responders in different risk score groups in the pediatric peptide vaccine immunotherapy cohort. **(D)** Proportion of immunotherapy responders in different risk score groups in the GBM anti-PD-1 immunotherapy cohort. **(E)** ROC curve assessing the predictive accuracy of risk score for immunotherapy response in the pediatric peptide vaccine immunotherapy cohort. **(F)** ROC curve assessing the predictive accuracy of risk score for immunotherapy response in the GBM anti-PD-1 immunotherapy cohort. **(G-I)** Analysis of risk score and sensitivity to various antineoplastic agents (Temozolomide, Gemcitabine, and Gefitinib), indicating differences in IC50 values across high and low risk score groups. (** p < 0.01; *** p < 0.001.).

The Tumor Immune Dysfunction and Exclusion (TIDE) algorithm evaluates the potential for tumor immune escape by analyzing gene expression profiles of tumor samples. Our analysis revealed that in the TCGA cohort, the high risk score group exhibited higher TIDE, Exclusion, and Dysfunction scores, along with lower MSI (microsatellite instability) scores. This suggests that patients in the high risk score group are more likely to experience immune escape and may have a poorer response to immune checkpoint inhibitor (ICI) therapy (**Figure 8A**). Conversely, the low risk score group predicted a higher proportion of patients likely to respond positively to immunotherapy (**Figure 8B**). These findings indicate that patients with a low risk score may derive greater benefit from immunotherapy compared to those with a high risk score.

In the LGG pediatric peptide vaccine immunotherapy cohort, the high risk score group predicted a higher proportion of patients responding to immunotherapy (**Figure 8C**). In contrast, in the GBM anti-PD-1 immunotherapy cohort, the low risk score group indicated a higher proportion of patients responding to immunotherapy (**Figure 8D**).The ROC of risk score predicting immunotherapy response was 0.77 and 0.626 in the two cohorts, respectively (**Figures 8E, F**).

Furthermore, we examined the relationship between risk score and drug therapy effectiveness in LGG treatment. Our findings revealed that high risk score values were associated with lower IC50 of Temozolomide and Gemcitabine, and higher IC50 of Gefitinib (**Figures 8G–I**, P < 0.05). Consequently, our study suggests that risk score could serve as a valuable predictor of drug therapy sensitivity in LGG patients.

### 3.6 Prognostic difference of drug treatment in different risk score groups

**Figure 9:**
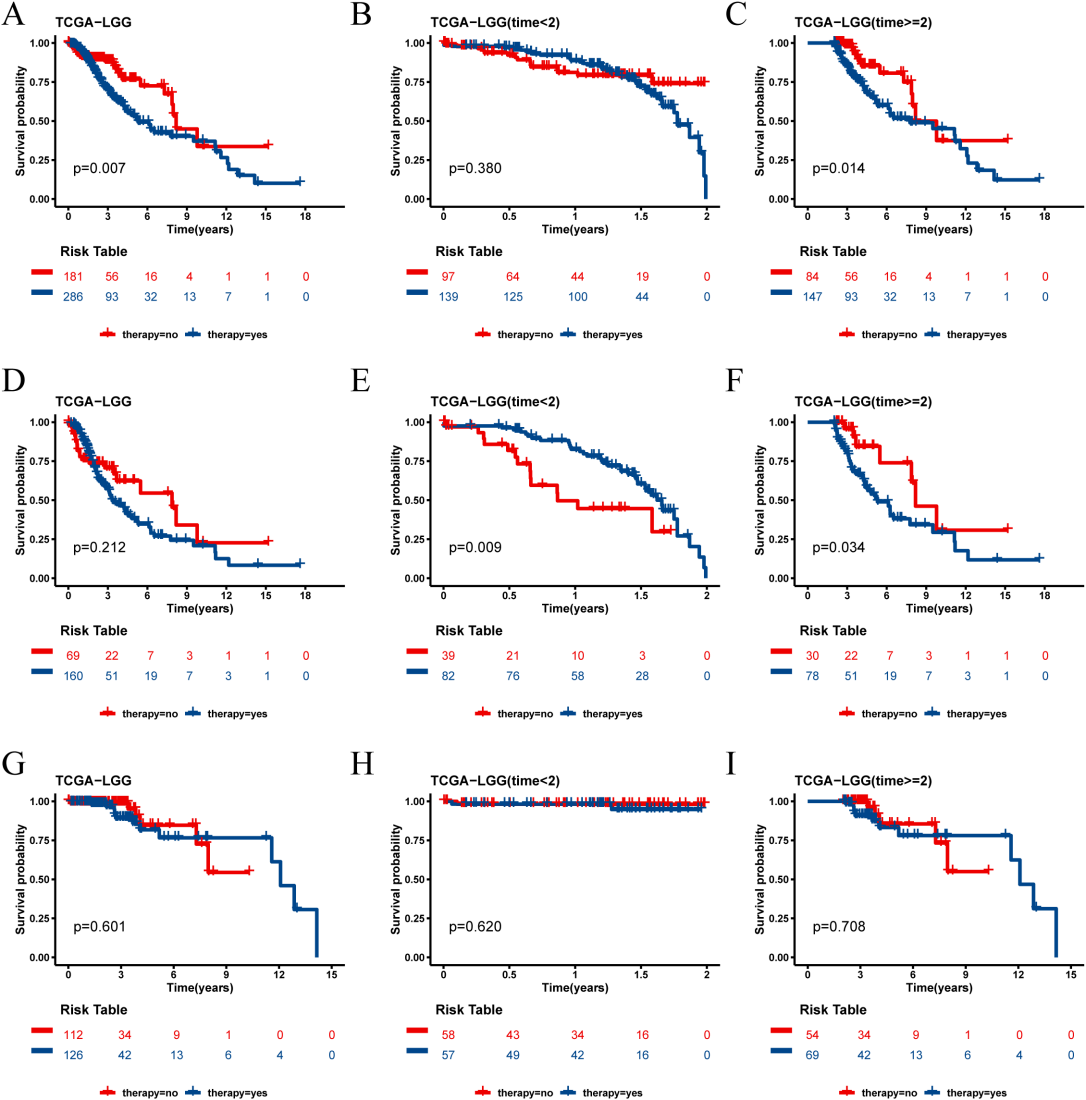
Survival analysis of TCGA cohort drug group and non-drug group. **(A)** Kaplan-Meier survival curves for all patients with LGG in the drug and non-drug groups. **(B)** Kaplan-Meier survival curves for short-term survival (< 2 years) in the drug and non-drug groups. **(C)** Kaplan-Meier survival curves for long-term survival (≥ 2 years) in the drug and non-drug groups. **(D)** Kaplan-Meier survival curves for all patients in the high risk group of LGG in the drug and non-drug groups. **(E)** Kaplan-Meier survival curves for short-term survival (< 2 years) in the high risk group of LGG in the drug and non-drug groups. **(F)** Kaplan-Meier survival curves for long-term survival (≥ 2 years) in the high risk group of LGG in the drug and non-drug groups. **(G)** Kaplan-Meier survival curves for all patients in the low risk group of LGG in the drug and non-drug groups. **(H)** Kaplan-Meier survival curves for short-term survival (< 2 years) in the low risk group of LGG in the drug and non-drug groups. **(I)** Kaplan-Meier survival curves for long-term survival (≥ 2 years) in the low risk group of LGG in the drug and non-drug groups.

In the TCGA-LGG cohort, we analyzed the survival outcomes of patients receiving medication versus those not receiving medication. At the 2-year survival time point, we observed distinct trends for short-term (< 2 years) and long-term (≥ 2 years) prognosis between the two groups (**Figures 9A–C**). Specifically, among all LGG patients, those receiving medication had a better short-term prognosis, while those not receiving medication had a better long-term prognosis.

Further analysis revealed that this phenomenon was particularly pronounced in the high risk score group. In this group, patients receiving medication had a better short-term prognosis but a worse long-term prognosis (**Figures 9D–F**). Conversely, in the low risk score group, the prognosis was not significantly affected by whether the patients received medication (**Figures 9H–J**).

The independent validation cohort CGGA-325 yielded results consistent with those of the TCGA-LGG cohort, reinforcing these observations (**Supplementary Figure 3**). These findings suggest that the risk score not only serves as a prognostic indicator but may also help identify which patients are likely to benefit from pharmacological treatment in the short term and require careful consideration for long-term prognosis. However, consistent results were not observed in the CGGA-693 cohort, and further studies are needed to validate these findings(**Supplementary Figure 4**).

## 4 Discussion

Although LGG is less malignant, its treatment still presents numerous challenges. The slow growth and poorly defined boundaries of LGG often make complete tumor resection difficult. Additionally, the effects of radiotherapy and chemotherapy on LGG can vary, with some patients developing resistance to these treatments, resulting in unsatisfactory outcomes. Even with a combination of surgical resection, radiotherapy, and chemotherapy, some patients still experience poor prognosis and high tumor recurrence rates. Furthermore, patients with LGG often face declines in cognitive function and quality of life during long-term treatment, making the management of treatment side effects a significant issue (29). Given the complexity and individual differences in LGG treatment, developing a reliable prognostic model is particularly important.

In this context, glycosylation, a common post-translational modification of proteins, has become the focus of our research. Glycosylation plays a crucial role in assisting immune cells with proper localization and migration (30). However, aberrant glycosylation modifications are closely associated with tumorigenesis, proliferation, invasion, metastasis, and immune escape (31, 32). Genetic, epigenetic, metabolic, inflammatory, and environmental mechanisms can lead to modifications of glycosylation, driving several biological processes in cancer (33). The first report of aberrant glycosylation modifications in tumors dates back 50 years (34). Due to the susceptibility to glycosylation, even minor pathogenic alterations or metabolic stress can lead to glycosylation dysfunction, resulting in aberrant sugar chains and glycoproteins (15). Understanding the causes and consequences of glycosylation changes associated with neoplastic disease will provide valuable insights into tumor development (31).

Risk models constructed from glycosylation-related genes have been shown to be closely related to overall survival and tumor microenvironment in patients with prostate cancer (14), lung adenocarcinoma (35), renal cell carcinoma (36), and bladder cancer (37). Risk models of N-glycosylation-related genes can effectively predict the prognosis of patients with hepatocellular carcinoma and the immune status of the tumor microenvironment (38). Despite this, the characterization of glycosylation-related gene sets in gliomas has not been comprehensively analyzed, and the relationship between glycosylation-related genes and glioma prognosis has been rarely studied.

Notably, a recent study showed that the prognostic model constructed by analyzing the differential glycosylation-related regulatory genes between glioma and normal brain tissue can accurately predict the prognosis of glioma patients. This is helpful in studying the occurrence and progression of glioma and identifying new targets for glioma diagnosis and treatment (39). However, due to the failure to properly stratify the samples according to the different grades of glioma in the study, this may affect the accurate revelation of gene expression differences of different grades of tumors and their correlation with prognosis.

Our study started from the single-cell level, identifying four cell types in LGG scRNA-seq data and finding that the glycosylation pathway is active in the microglial population. Combined with TCGA-LGG data, we constructed and validated glycosylation-related risk characteristics based on eight glycosylation-related genes (ASPM, CHI3L1, LILRA4, MSN, OCIAD2, PTGER4, SERPING1, and TNFRSF12A). Among the eight glycosylation-related genes we studied, ASPM (Abnormal spindle-like microcephaly associated protein) is a spindle pole/intermediate protein that regulates mitosis and cytoplasmic division (40). ASPM is aberrantly expressed in various tumors, such as glioblastoma (41), endometrial adenocarcinoma (42), pancreatic cancer (43), prostate adenocarcinoma (44), and ovarian cancer (45, 46), and is associated with tumor prognosis. CHI3L1 (chitinase-3-like protein 1) is a member of the glycoside hydrolase family (47). Elevated serum CHI3L1 levels correlate with disease severity in a variety of human tumors, including breast, colon, prostate, ovarian, brain, thyroid, lung, and liver cancers, leading to poorer prognosis and shorter survival (48). In gliomas, CHI3L1 reprograms the tumor microenvironment (TME) by promoting NF-κB pathway activation, regulating tumor malignancy and local invasiveness, making it a potential therapeutic target for gliomas (49, 50). Dendritic cells (DCs) are divided into myeloid dendritic cells (mDCs) and plasmacytoid dendritic cells (pDCs) (51), with pDCs specifically expressing the orphan receptor immunoglobulin-like transcript 7 (ILT7, also known as LILRA4 and CD85g) (52). Typically, ILT7/ILT7L signaling produces a negative immune response feedback following viral infection (53). Intervention in the ILT7L/ILT7 system may enhance anti-tumor and antiviral immunity (51). The Moesin protein encoded by the MSN gene is part of the ezrin-radixin-moesin (ERM) protein family (54). Moesin is upregulated in various human cancers, including breast cancer, prostate cancer, pancreatic cancer, lung cancer, and melanoma (55). Studies suggest that MSN could be a novel therapeutic target for colorectal cancer (56). OCIAD2, part of the ovarian cancer immune response antigen (OCIA) domain family, promotes tumor metastasis by enhancing STAT3 activation and cell migration (57). OCIAD2 has been associated with prognosis in bladder cancer patients and shows potential in immunotherapy (58). The methylation status of OCIAD2 may be a useful prognostic indicator in patients with hepatoblastoma (59) and lung adenocarcinoma (60). PTGER4 is a major prostaglandin E2 (PGE2) receptor whose genetic variation and expression levels can affect gastric cancer (61). SERPING1 encodes a highly glycosylated plasma protein involved in the regulation of the complement cascade and immune responses (62). Studies indicate that SERPING1 can serve as a novel marker for prostate cancer diagnosis and prognosis (63) and is relevant for early detection of bone metastases in breast cancer (64). Fibroblast growth factor inducible 14 (Fn14; TNFRSF12A) is a cell surface receptor for TNF-like weak inducers of apoptosis (TWEAK), part of the tumor necrosis factor (TNF) family. TNFRSF12A expression is usually low in normal tissues but significantly increases after tissue injury and in many solid tumor types (65), including glioma, breast cancer, esophageal adenocarcinoma, pancreatic cancer, and hepatocellular carcinoma. Overexpression of TNFRSF12A is associated with poor prognosis in these tumors (66).

The resulting risk score as a novel prognostic biomarker in patients with LGG, can predict ICB immunotherapy response. TIDE analysis demonstrated that patients with a low risk score had a higher immunotherapy response rate compared to those with a high risk score. In the external immunotherapy cohort, the results of the GBM anti-PD-1 immunotherapy cohort supported this finding, while the LGG pediatric peptide vaccine immunotherapy cohort showed a higher proportion of patients predicted to respond to immunotherapy in the high risk score group due to the smaller sample. And the data of these two cohorts indicated that risk score can be used to predict immunotherapy response. Additionally, we explored the relationship between risk characteristics, immune characteristics, and drug sensitivity. Our results suggested that patients in the high risk score group exhibit higher glycosylation pathway activity and a more complex immune microenvironment compared to those in the low risk score group, while the high risk score was associated with lower IC50 for Temozolomide and Gemcitabine, as well as higher IC50 for Gefitinib. Innovatively, we discovered that LGG patients treated with drugs tend to have a better short-term prognosis but a poorer long-term prognosis. Conversely, patients with LGG who do not receive drug treatment have a good long-term prognosis but a poor short-term prognosis. And this difference in prognosis is particularly significant in the high risk score group. This finding provides new insights into the treatment strategy for LGG.

By evaluating the predictive value of these genes in the prognostic model for LGG patients, we have revealed their potential roles in the effectiveness of immunotherapy and drug therapy, providing a basis for precise treatment of LGG patients. However, the study has some limitations. First, the scRNA-seq data involves a limited number of samples, which may affect the accuracy and reliability of the results. And there is a lack of higher quality immunotherapy cohorts to validate the predictive power of risk score in predicting immunotherapy response. Second, further studies are needed to validate the roles of these genes in the development of LGG and to understand their potential mechanisms and therapeutic value.

To overcome these limitations, we plan to conduct more in-depth studies with larger sample sizes to further validate and expand upon these findings. We hope that these follow-up studies will provide more effective treatment strategies and prognostic evaluation tools for LGG patients, thereby improving their overall prognosis and quality of life.

## 5 Conclusion

Glycosylation are pivotal in tumor biology, influencing tumor development and impacting the immune microenvironment of LGG. The risk features based on glycosylation constructed and validated in this study are robust predictors of overall survival (OS) in LGG patients. Importantly, these features also hold promise for predicting response to immunotherapy. Our study contributes a novel perspective by unraveling the mechanisms underlying LGG prognosis and offering insights into potential avenues for personalized cancer immunotherapy.

## Data Availability

The original contributions presented in the study are included in the article/**Supplementary Material**. Further inquiries can be directed to the corresponding authors.
