# Supplementary material for: Application of a risk score model based on glycosylation-related genes in the prognosis and treatment of patients with low-grade glioma
Source: Front Immunol. 2024 Oct 9;15:1467858. doi: 10.3389/fimmu.2024.1467858 (PMC11496118; doi:10.3389/fimmu.2024.1467858)
Supplement: Supplementary file 1 [file DataSheet1.docx]

Supplementary Material

## Supplementary Figures

###
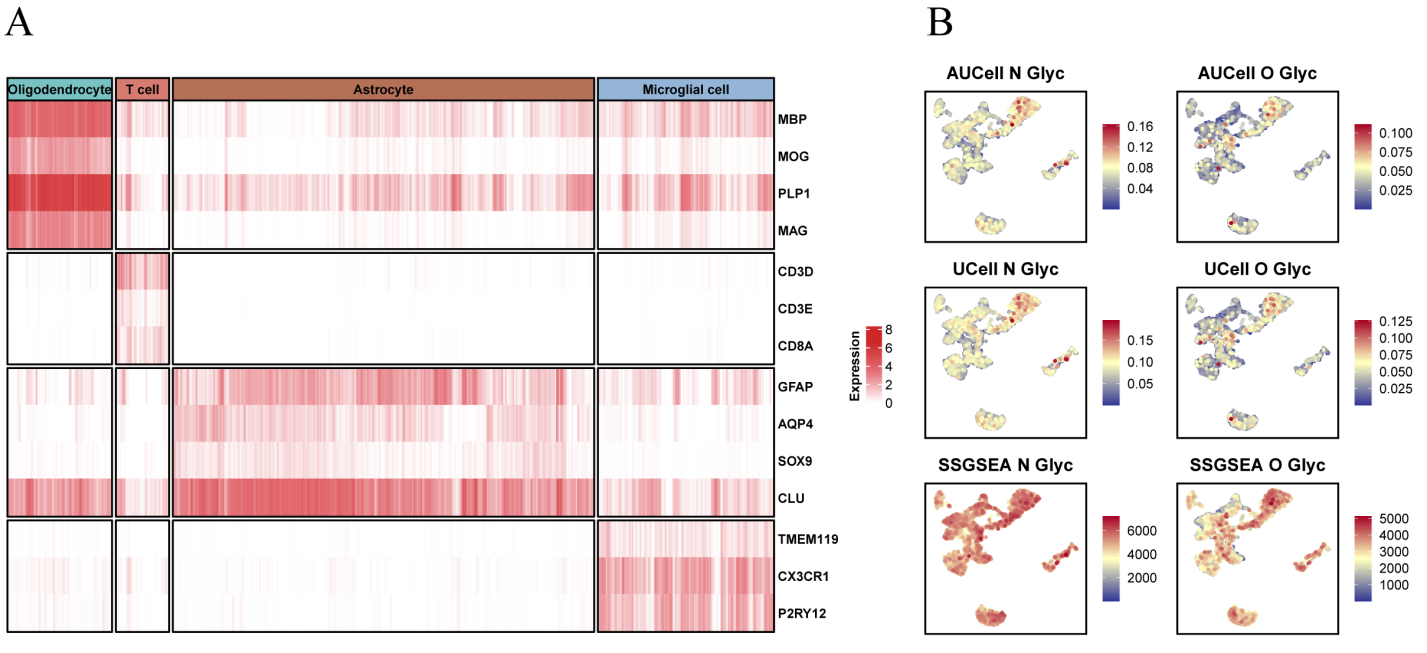


**Supplementary Figure 1.** Distribution of marker and glycosylation scores in single-cell RNA sequencing of glioma. **A.** Heatmap showing the expression levels of marker genes in oligodendrocytes, T cells, astrocytes, and microglial cells. **B**. UMAP plots showing the distribution of N-glycosylation and O-glycosylation pathway activity scores across cell types using different scoring methods (AUCell, UCell, and SSGSEA).


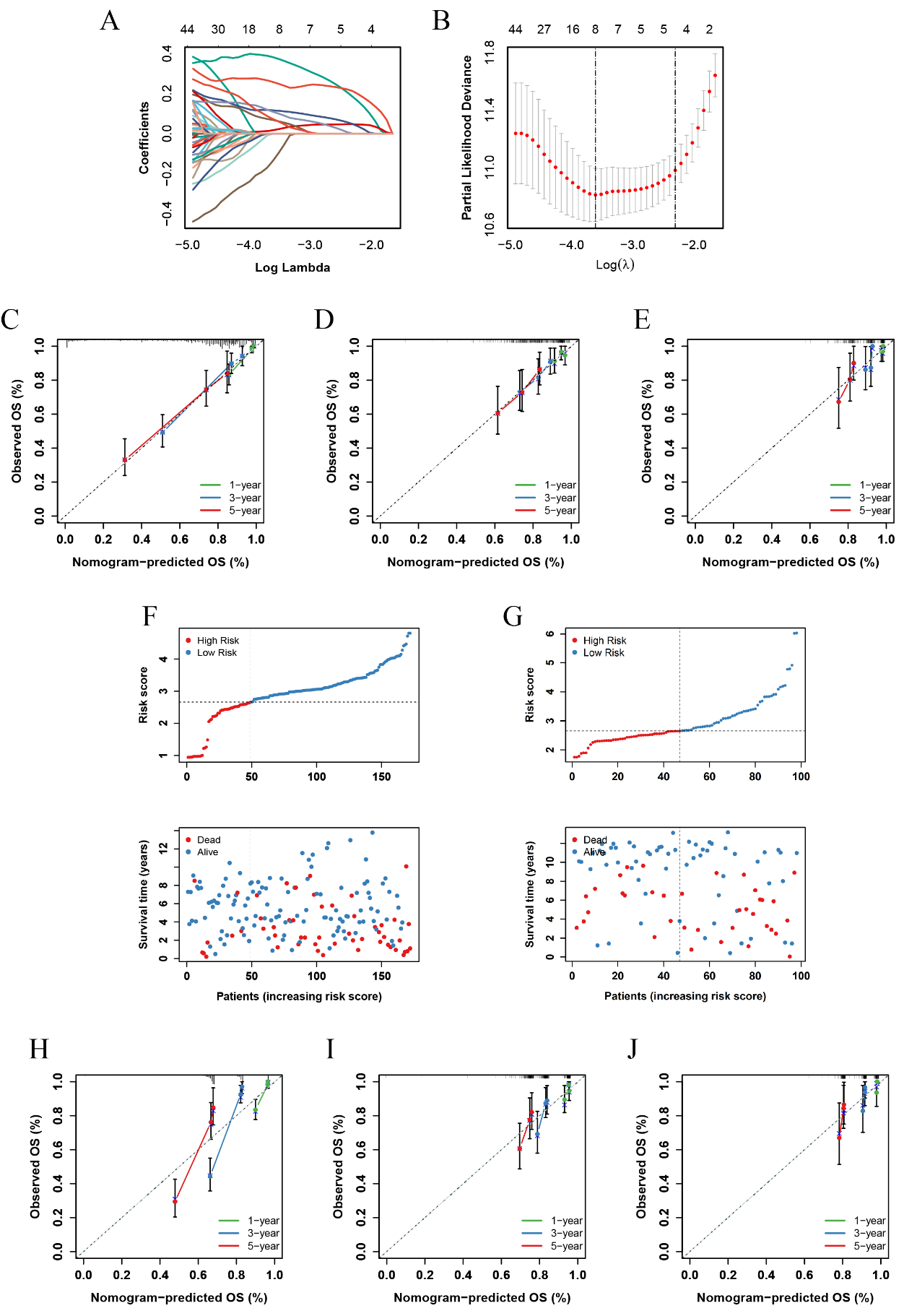


**Supplementary Figure 2.** Establishment and validation of risk score and nomogram. **A-B.** Eight prognostic DEGs were identified by LASSO regression analysis after univariate Cox regression analysis. **C-E.** Risk score calibration curves for three cohorts (TCGA-LGG, CGGA-LGG693, CGGA-LGG325) demonstrating accuracy in predicting survival. **F-G.** Distribution of risk score for patients with LGG in the CGGA-LGG693 and CGGA-LGG325 cohorts, ranked from lowest to highest. The survival status of each patient with LGG was classified by risk score. **H-J.** Nomogram model calibration curves for three cohorts (TCGA-LGG, CGGA-LGG693, CGGA-LGG325).


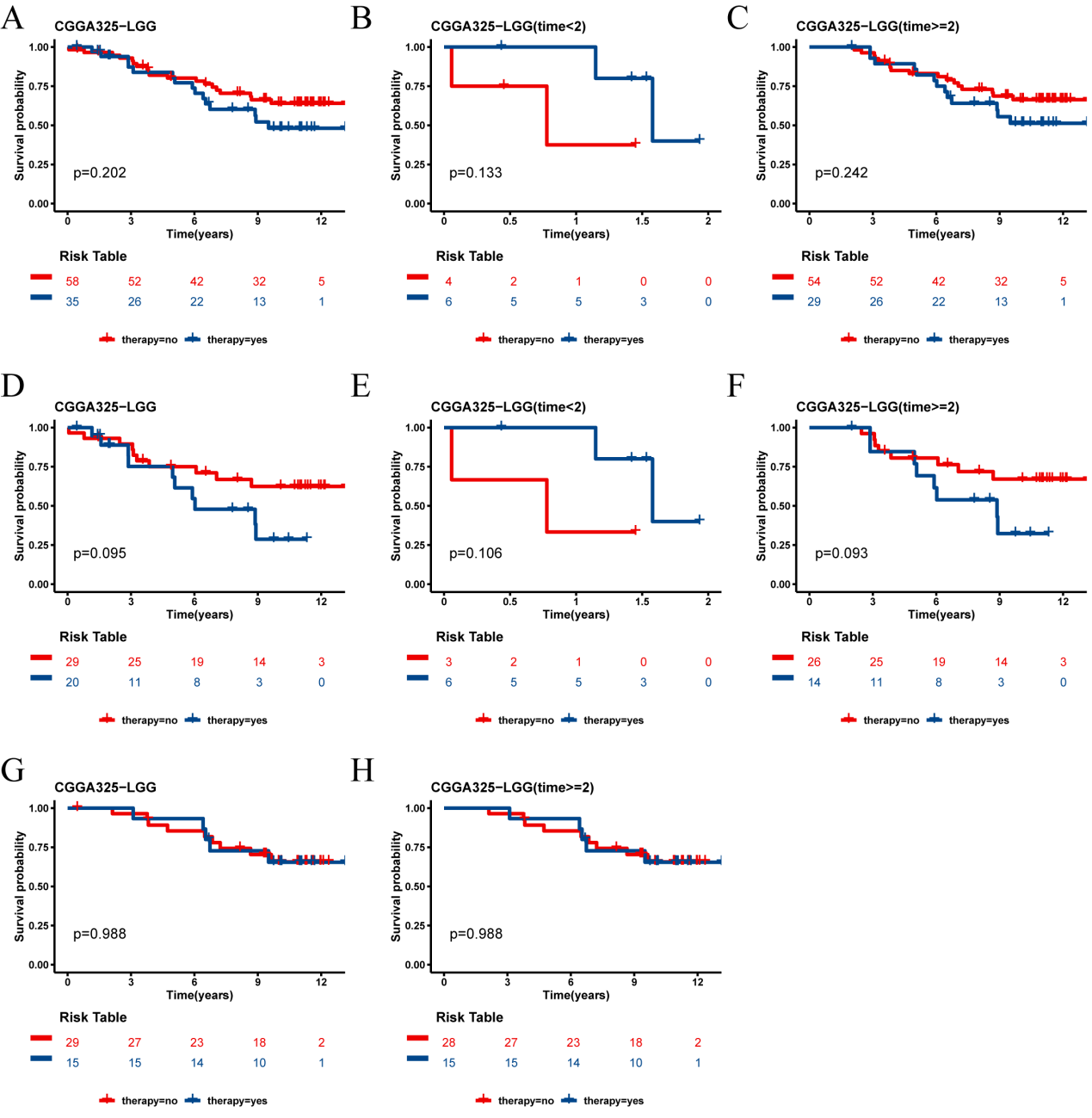


**Supplementary Figure 3.** Survival Analysis of CGGA-LGG325 Cohort Drug Group and Non-Drug Group **A.** Kaplan-Meier survival curves for all patients with LGG in the drug and non-drug groups. **B**. Kaplan-Meier survival curves for short-term survival (< 2 years) in the drug and non-drug groups. **C.** Kaplan-Meier survival curves for long-term survival (≥ 2 years) in the drug and non-drug groups. **D.** Kaplan-Meier survival curves for all patients in the high risk group of LGG in the drug and non-drug groups. **E.** Kaplan-Meier survival curves for short-term survival (< 2 years) in the high risk group of LGG in the drug and non-drug groups. **F.**  Kaplan-Meier survival curves for long-term survival (≥ 2 years) in the high risk group of LGG in the drug and non-drug groups. **G.** Kaplan-Meier survival curves for all patients in the low risk group of LGG in the drug and non-drug groups. **H.** Kaplan-Meier survival curves for long-term survival (≥ 2 years) in the low risk group of LGG in the drug and non-drug groups.


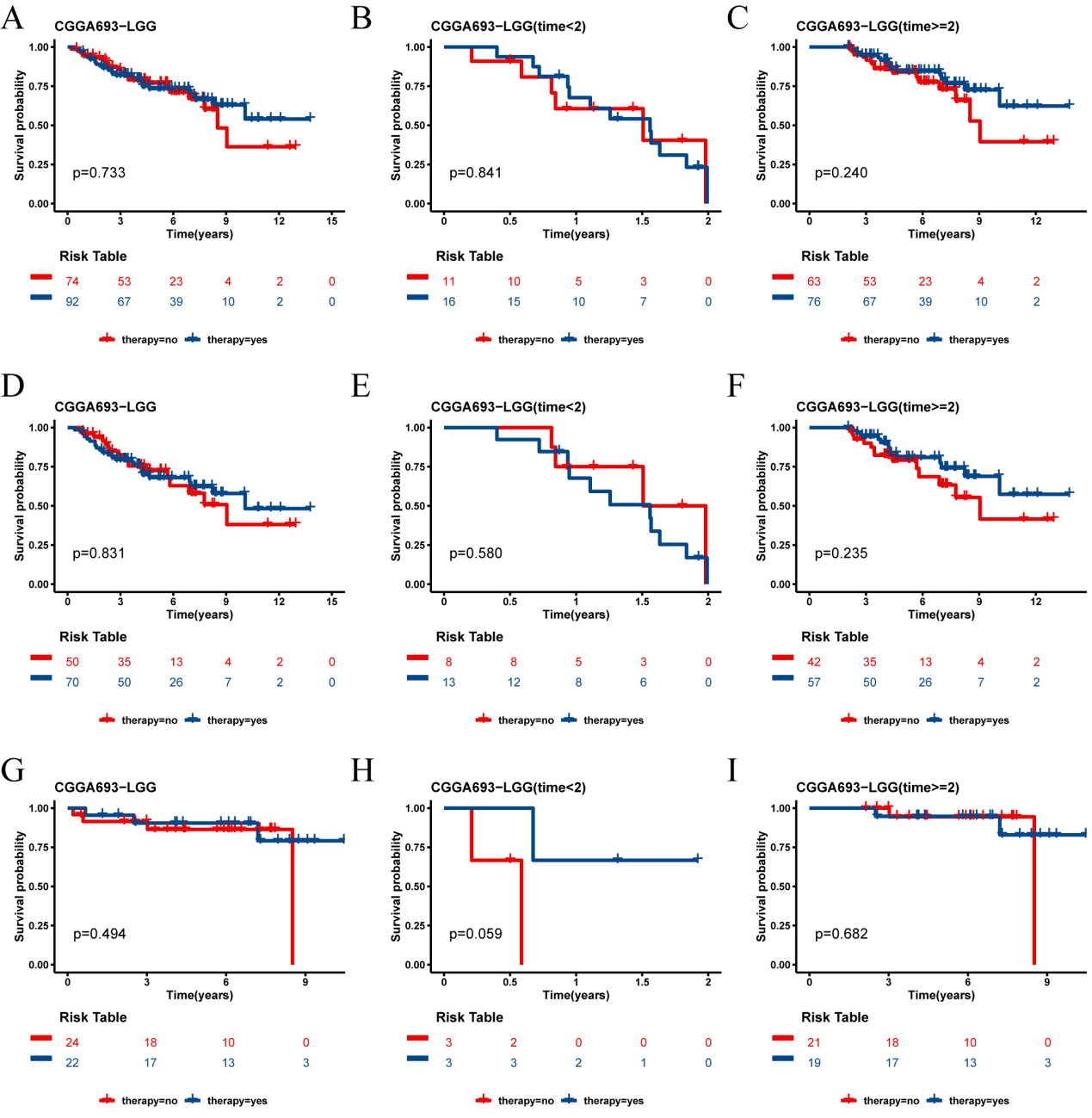


**Supplementary Figure 4.** Survival Analysis of CGGA-LGG693 Cohort Drug Group and Non-Drug Group **A.** Kaplan-Meier survival curves for all patients with LGG in the drug and non-drug groups. **B**. Kaplan-Meier survival curves for short-term survival (< 2 years) in the drug and non-drug groups. **C.** Kaplan-Meier survival curves for long-term survival (≥ 2 years) in the drug and non-drug groups. **D.** Kaplan-Meier survival curves for all patients in the high risk group of LGG in the drug and non-drug groups. **E.** Kaplan-Meier survival curves for short-term survival (< 2 years) in the high risk group of LGG in the drug and non-drug groups. **F.**  Kaplan-Meier survival curves for long-term survival (≥ 2 years) in the high risk group of LGG in the drug and non-drug groups. **G.** Kaplan-Meier survival curves for all patients in the low risk group of LGG in the drug and non-drug groups. **H.** Kaplan-Meier survival curves for short-term survival (< 2 years) in the low risk group of LGG in the drug and non-drug groups. **I.** Kaplan-Meier survival curves for long-term survival (≥ 2 years) in the low risk group of LGG in the drug and non-drug groups.
